# Breaking the acoustic diffraction limit with an arbitrary shape acoustic magnifying lens

**DOI:** 10.1038/s41598-021-92297-7

**Published:** 2021-06-21

**Authors:** Ali Abdolali, Hooman Barati Sedeh, Mohammad Hosein Fakheri, Chen Shen, Fei Sun

**Affiliations:** 1 Applied Electromagnetic Laboratory, School of Electrical Engineering, Iran University of Science and Technology, 1684613114 Tehran, Iran; 2 Department of Mechanical Engineering, Rowan University, 201 Mullica Hill Road, Glassboro, NJ 08028 USA; 3 College of Physics and Optoelectronics, Taiyuan University of Technology, Taiyuan, 030024 China

**Keywords:** Acoustics, Transformation optics, Metamaterials

## Abstract

Based on the transformation acoustics methodology, the design principle for achieving an arbitrary shape magnifying lens (ASML) is proposed. Contrary to the previous works, the presented ASML is competent of realizing far-field high resolution images and breaking the diffraction limit, regardless of the position of the utilized sources. Therefore, objects locating within the designed ASML can be properly resolved in the far-field region. It is shown that the obtained material through the theoretical investigations becomes an acoustic null medium (ANM), which has recently gained a significant attention. Besides the homogeneity of ANM, which makes it an implementable material, it is also independent of the perturbation in the geometry of the lens, in such a way that the same ANM can be used for different structural topologies. The obtained ANM has been implemented via acoustics unit cells formed by membranes and side branches with open ends and then was utilized to realize an ASML with the aid of effective medium theory. It is shown that the far-field results of an ideal ASML abide well with the results of the implemented sample, validating the proposed design principle. The presented acoustic magnifying lens has a wide spectrum of possible applications ranging from medical imaging, and biomedical sensors to focused ultrasound surgery.

## Introduction

Imaging with the acoustic waves is one of the most widely used methods in applications such as industrial test and clinical diagnosis. However, the resolution of conventional imaging systems is inherently restricted by the diffraction limit, as the information carried by evanescent waves cannot be effectively delivered to the far field^1^. To obviate this limit, acoustic super-lenses (ASL) have been proposed that are capable of achieving sub-diffraction-limit by partially magnifying evanescent waves^2,3^. However, ASL usually requires meta-atoms with highly dispersive characteristics, which are difficult to be realized in practice and are not suitable for broadband applications. To overcome the narrowband nature of ASL, acoustic hyper-lens (AHL) was propounded as an alternative for converting evanescent components associated with the deep sub-wavelength into propagating waves and; thus, making it possible to break the diffraction limit over a broad frequency bandwidth^4,5^. Nevertheless, AHLs are significantly sensitive to the position of the sources, implying that only objects located close to the circumference of the lens can be properly resolved. This has also been verified experimentally by Li et al.^6^ as only sources locating in the close vicinity of the input surface of the designed AHL can be appropriately resolved on the output interface. This inherent nature of the AHLs will consequently limit the applicability of such lenses for being used in practical scenarios. In addition to the location of the sources, to date, all the propounded works are merely limited to two specific geometries of cylindrical and planar, which consequently restrict the applicability of these lenses for being used in more general situations. Moreover, owing to the fixed functionality of these lenses, their corresponding magnification factors are set to stone after their implementation and cannot be altered afterward^5,7–10^. Therefore, achieving an acoustic magnifying lens (AML) that can overcome the aforementioned problems is still a challenge which needs to be addressed. Besides material engineering, other approaches such as the utilization of core-shell-shaped lenses have been propounded to overcome the ultrasonic diffraction limit. However, despite the material simplicity and feasible implementation, the obtained resolution is limited compared to the aforementioned techniques^11–13^.

Transformation acoustics (TA), which its underlying physics comes from the well-established method of coordinate transformation^14–23^, provides a set of powerful design tools that can be used for the creation of devices with an unprecedented ability to manipulate acoustic waves^24–26^. Following this approach, a new route to manipulate acoustic waves for high-resolution imaging and achieving AML has been opened recently^27^. However, the main challenge of TA-based AMLs is their inhomogeneous constructive materials that cause difficulties for their fabrication in real-life scenarios. Moreover, the same as other conventional approaches, TA-based lenses are shape dependent which leads the applicability of these platforms to be limited to particular scenarios^5,28,29^. Here, on the basis of coordinate transformation method, we will introduce the design principle of an arbitrary shape magnifying lens (ASML) that can efficiently transfer the acoustic sub-wavelength details from objects to far field region and thus breaking the diffraction limit. In particular, we will establish a rigorous theoretical formulation based on our recent work^26^ which leads to a transformation function that maps a region with a short radius to another domain with a longer radii. As a result of such a transformation, all the points within the smaller region are magnified by the  ratio of two radii, a parameter that has been dubbed as magnification factor throughout the paper. Then, we will obtain a specific kind of material, known as acoustic null medium (ANM)^26,30–32^, that when it surrounds the utilized sources (locating at a subwavelength distance), the total scattered field mimic as if the sources were located in a distance which is not subwavelength. The main advantage of the proposed method is its resultant materials which are homogeneous and independent of the the lens structural topology, indicating that regardless of the lens shape, one can use the same materials for different geometries. Several numerical simulations were performed in order to verify the effectiveness of the proposed ASML. Although previously the ANM has been designed with the aid of effective medium theory (EMT)^33^ together with exploiting natural materials, assuming tungsten hexafluoride as a background medium restricts its application in real-life scenarios. In particular, in our recent work^26^, we have assumed the background medium to be filled with a particular material, that in general is not always possible and as such it will restrict the applicability of the realized ANM. Therefore, to fill the long-standing gap between the theory of ASML and its design in the context of acoustic metamaterials, here, we have realized the obtained ANM by using nonresonant acoustic meta-atoms made of periodic cubic blocks with clamped elastic membranes and side branches, which compared to our recent publication, not only is more practical, as one can simply change its geometrical parameters to obtain the desired characteristics, but also is more simple and cost-effective. It is shown that by changing the thickness of the membrane and geometry of the side branches, one can achieve the desired density and bulk modulus of the ANM. Then, it is demonstrated that the realized ANM with the proposed metamaterial is competent of mimicking the behavior of an ideal ANM and thus applicable to be used for implementing an ASML. Finally, as a proof of concept, a practical and realistic ASML is designed and implemented with multi-layered structure made of the realized ANM. It is observed that the image of the utilized sources which were located in close vicinity of each other is properly resolved. The proposed ASML represents a significant step toward realizing super-resolution imaging devices that may greatly benefit the development of new-concept acoustic imaging and detection systems in the future.

## Results

### Theory

**Figure 1:**
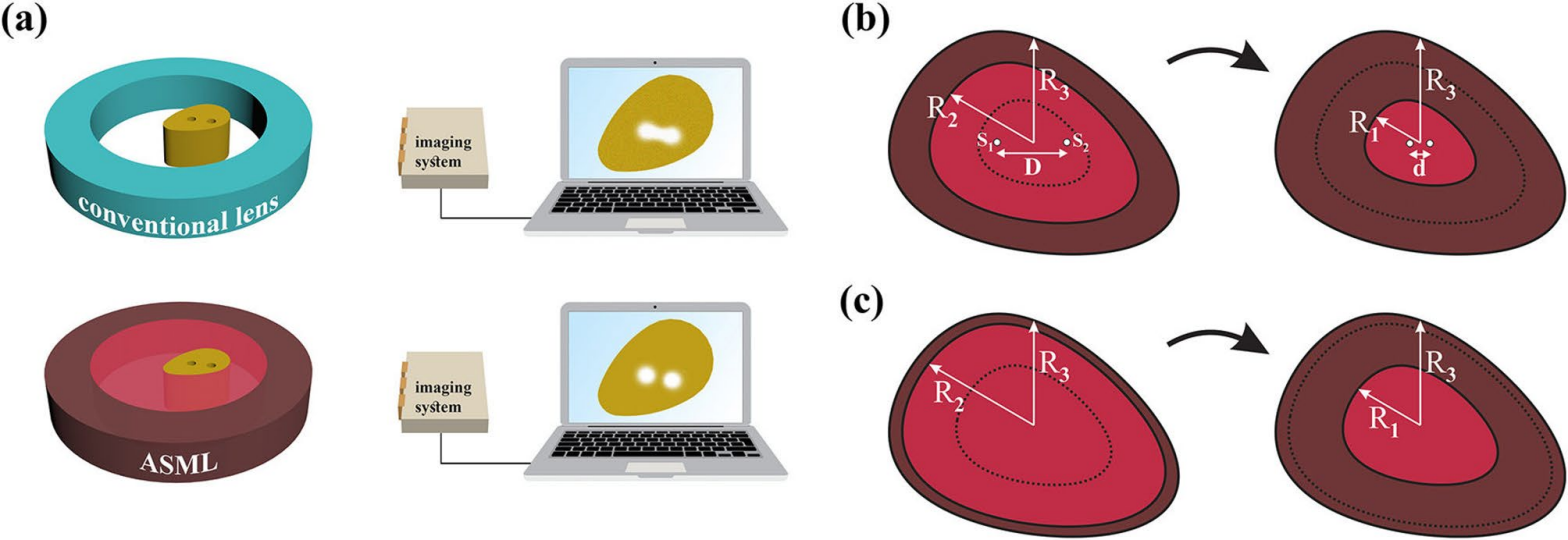
(**a**) The schematic of potential application of ASML in sub-wavelength imaging devices. (**b**) The demonstration of the coordinate transformation. (**c**) The corresponding coordinate transformation when $h_2 \rightarrow h_3$.

The schematic of potential application of the ASML in a imaging system is illustrated in Fig. 1a. As can be seen, the image sources which are two holes that are separated by a sub-wavelength distance of *d* are placed on a plate and excited by an acoustic plane wave. Since these holes are located in close vicinity of each other, their images cannot be resolved in the far-field region due to the diffraction limit^34,35^. The goal is to design a coating layer on the basis of coordinate transformation methodology that is capable of obviating the diffraction problem and giving rise to two distinguished images in the far-field region. To this aim, the inner red region in the physical space, $r{^\prime} < R_1(\varphi )$ (compressed domain), must be extended into the larger domain in the virtual space, $r < R_2(\varphi )$ , while at the meantime, the brown region of $R_1(\varphi )< r{^\prime} < R_3 (\varphi )$ (stretched domain) should be compressed into the domain of $R_2 (\varphi )< r < R_3 (\varphi )$ as shown in Fig. 1b. As a result, for an outside observer, all the points within the smaller region are magnified and consequently, the total scattered field behaves as if the holes were located in a distance that is not subwavelength. The mathematical expression that will lead to such a mapping could be written as

1 $$\begin{aligned} \ \left\{ \begin{array}{lll} r_1{^\prime} =\frac{R_1(\varphi )}{R_2(\varphi )}r&{} r{^\prime} \in [0, R_1(\varphi ))\\ r_2{^\prime} =\frac{R_3(\varphi )-R_1(\varphi )}{R_3(\varphi )-R_2(\varphi )} r + \frac{R_1(\varphi )-R_2(\varphi )}{R_3(\varphi )-R_2(\varphi )} R_3(\varphi ) &{} r{^\prime} \in [R_1(\varphi ), R_3(\varphi )) \\ r_3{^\prime} = r &{} r{^\prime} \in [R_3(\varphi ),\infty ) \end{array} \right. \ \end{aligned}$$

Under the transformation of Eq. (1), the sub-wavelength distance (i.e., *d*) between two holes (i.e., $S_1$ and $S_2$ in Fig. 1b) within the physical space is magnified to distance *D* in the virtual space^8,36^. In other words, although the acoustic holes are located in close vicinity of one another, an outside observer will perceive two holes that are separated with distance *D* between them and as a result, the details of the object could be resolved from the obtained images. To measure the amount of magnification, it is convenient to introduce the magnification factor as $\Gamma =D/d$. On the other hand, according to Eq. (1), the magnification factor can be related to the topological structure of the ASML with the relation of $\Gamma =D/d=R_2(\varphi )/R_1(\varphi )$, indicating that by changing the contour shapes, one can achieve the desired magnification factor. However, the main drawback of such an ASML is its obtained inhomogenous and shape dependent materials.

According to the TA methodology^24^, the demanding materials after any coordinate transformation will be acheived as $\rho {^\prime} =\rho _0 \det (\Lambda )(\Lambda ^{-1})^T (\Lambda ^{-1})$ and $\kappa {^\prime} = \kappa _0 \det (\Lambda )$, where $\rho _0$ and $\kappa _0$ are the mass density and bulk modulus of the background medium, and $\Lambda = \partial (r{^\prime} ,\varphi {^\prime} ,z{^\prime} )/(r,\varphi ,z)$ is the Jacobian matrix that relates virtual space to the physical one. Therefore, following the transformation function given in Eq. (1), the demanding materials for such an ASML will be obtained as

2a $$\rho _{c}^{\prime } /\rho _{0} = \left[ {\begin{array}{*{20}c} 1 & 0 & 0 \\ 0 & 1 & 0 \\ 0 & 0 & {(R_{1} (\varphi )/R_{2} (\varphi ))^{2} } \\ \end{array} } \right],\;\kappa _{c}^{\prime } = \kappa _{0} (R_{1} (\varphi )/R_{2} (\varphi ))^{2} {\text{ }}$$

2b $$\rho _{s}^{\prime } /\rho _{0} = \left[ {\begin{array}{*{20}c} {\rho _{{11}} } & {\rho _{{12}} } & 0 \\ {\rho _{{21}} } & {\rho _{{22}} } & 0 \\ 0 & 0 & {\rho _{{33}} } \\ \end{array} } \right],\;\kappa _{s}^{\prime } = \kappa _{0} \frac{{r_{s}^{\prime } \times \partial r_{s}^{\prime } /\partial r}}{r}{\text{ }}$$

where subscripts of c and s represent the compressed and stretched region and $\rho _{ij}$, are defined as

3 $$\rho _{{11}} = \frac{{r_{2}^{\prime } }}{{r \times \partial r_{2}^{\prime } /\partial r}},\;\rho _{{12}} = \rho _{{21}} = - \frac{{\partial r_{2}^{\prime } /\partial \varphi }}{{r \times \partial r_{2}^{\prime } /\partial r}},\;\rho _{{22}} = \frac{{(\partial r_{2}^{\prime } /\partial \varphi )^{2} }}{{r \times r_{2}^{\prime } \times \partial r_{2}^{\prime } /\partial r}} + \frac{{r \times \partial r_{2}^{\prime } /\partial r}}{{r_{2}^{\prime } }},\;\rho _{{33}} = \frac{{r_{2}^{\prime } \times \partial r_{2}^{\prime } /\partial r}}{r}$$

According to Eqs. (1–3), any change in the contour shapes will result in new materials which must be redesigned and re-fabricated for each new case. Besides, since the magnification factor has a direct relation with the geometry of the ASML, having a reconfigurable $\Gamma$ deemed to be impossible via the proposed material. It should be emphasized that the magnification in here indicates the increase in the distance of two sources, which directly affects the resolution of the farfield pattern. In addition to the shape dependency of the obtained materials, they are also inhomogeneous and anisotropic with off-diagonal components in both regions, which cause serious difficulties for their realization. Nevertheless, as in practical scenarios only the geometry of the domain where the utilized sources are located within is important, one can assume the lens has conformal boundaries. From the mathematical point of view, this assumption can be written as $R_1(\varphi )/h_1=R_2(\varphi )/h_2=R_3(\varphi )/h_3=H(\varphi )$ where $h_i$ are constant coefficients and $H(\varphi )$ is the desired contour shape which can be defined by a truncated Fourier series^26^. By applying this assumption, the inhomogeneity of the obtained materials in the compressed region (i.e., $\rho _c{^\prime}$ and $\kappa _c{^\prime}$) will be obviated. Moreover, the magnification factor can also become reconfigurable, $\Gamma =h_2/h_1$, as one can easily change the size of the contours (i.e., $h_i$) while preserving its shape (i.e., $H(\varphi )$). Nevertheless, the materials of the stretched region will remain inhomogeneous and anisotropic due to the existence of $H(\varphi )$ in the second term of $r_2{^\prime}$. To overcome this issue, we will take the advantage of the fact that $R_2(\varphi )$ is in fact a fictitious region and thus can achieve any arbitrary value. In other words, regardless of the exact value of $h_2$, the ASML will exhibit perfect functionality as long as the condition of $h_1<h_2<h_3$ is satisfied. Therefore, without the loss of generality, one can assume ${{h}_{2}}\rightarrow {{h}_{3}}$ as it is shown in Fig. 1c. As it is discussed in the method section, this selection of value for $h_2$ will lead  the acoustic waves to be independent of $\rho _{12}$ and $\rho _{21}$, which gives us this degree of freedom to select the value of these components arbitrarily. In this paper, we have selected the off-diagonal components of Eq. (3) to be zero (i.e., $\rho _{12}=\rho _{21}=0$) that consequently leads the final material of the stretched and compressed domains to be changed to

4a $$\rho _{c}^{\prime } = \left[ {\begin{array}{*{20}c} 1 & 0 & 0 \\ 0 & 1 & 0 \\ 0 & 0 & {(1/\Gamma )^{2} } \\ \end{array} } \right],\;\kappa _{c}^{\prime } = (1/\Gamma )^{2} {\text{ }}$$

4b $$\rho _{s}^{\prime } = \left[ {\begin{array}{*{20}c} 0 & 0 & 0 \\ 0 & {1/\Delta } & 0 \\ 0 & 0 & {1/\Delta } \\ \end{array} } \right],\;\kappa _{s}^{\prime } = 1/\Delta {\text{ }}$$

where $\Delta$ is an arbitrary constant number that should satisfy the condition of $\Delta \rightarrow 0$. The obtained material of the stretched region in Eq. (4b) has been named as ANM^26^. Using Eq. (4a) for the compressed region around the sources located at the subwavelength distance, makes the acoustic fields on $R_1(\varphi ')$, to be the same as the acoustic fields on $R_3(\varphi )$ for the sources that are significantly apart. In the next step, with the aid of ANM presented in Eq. (4b), these fields will be point-to-point transmitted to its output surface (i.e., $R_3(\varphi ')$) without any distortion. As a result, the obtained materials, magnify the distance of the sources in such a way that the radiated fields mimic as if the sources were located in a distance that is not subwavelength. Moreover, as can be seen from Eq. (4b), the functionality of the ANM is independent of the device shape. That is if the structural geometry is changed, there is no need to recalculate and redesigned the previous materials. It should also be noted that to obtain the demanding material for the ASML we did not employ any restriction. However, depending on the desired magnification factor, the outer contour radius should be modified. It is noteworthy to mention that the selected value for the ratio of $R_3/R_1$, will directly affect the demanding material of its corresponding region as well as the magnification factor of the lens. When this ratio is constant, changing the contour shape of the device will not affect its corresponding materials.

**Figure 2:**
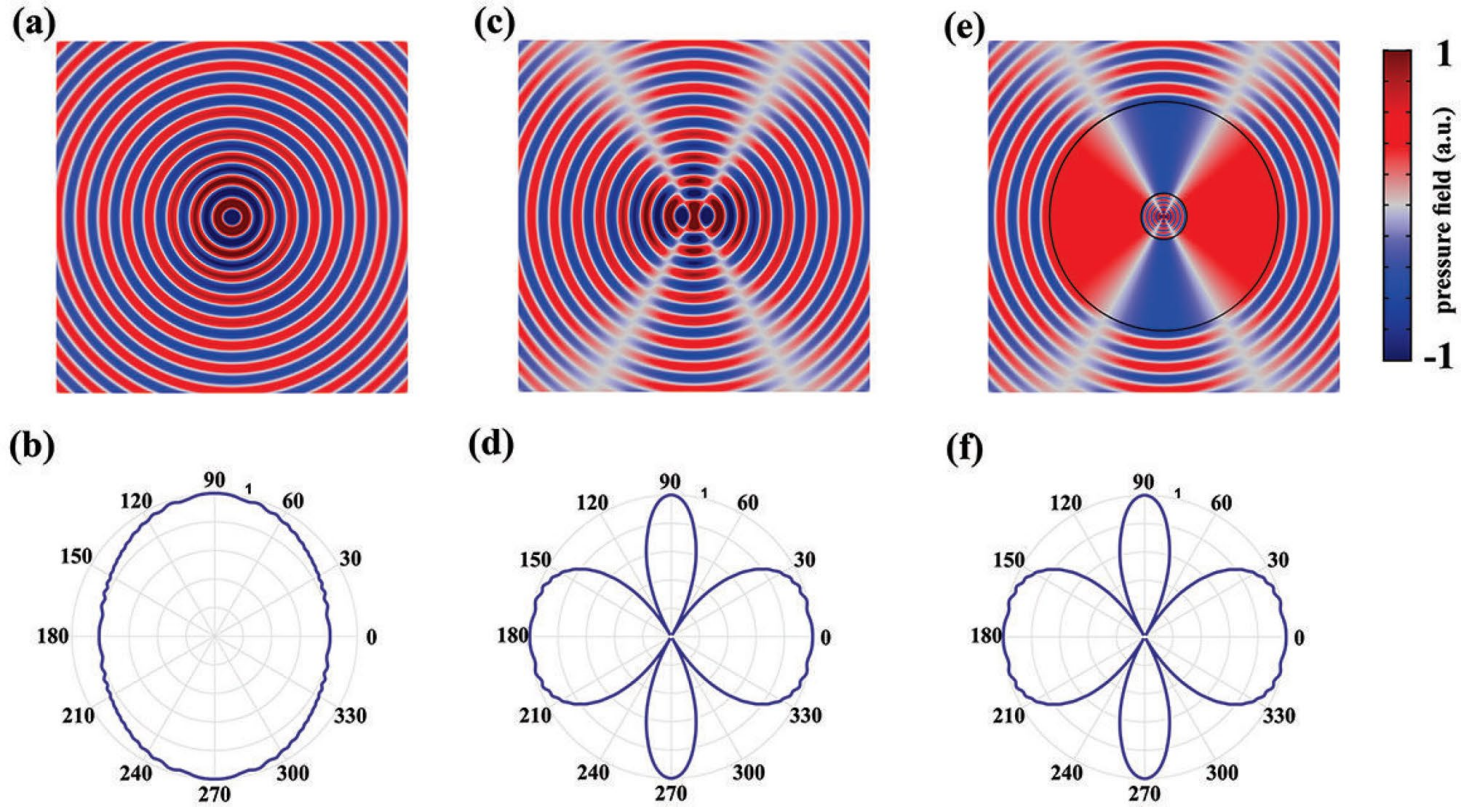
(**a**,**b**) The near and far field results of two line sources located at the sub-wavelength distance of $d=0.2 \lambda$. It is clear that the diffraction effect is significant in this case, which subsequently leads the farfield image to not be resolved appropriately. (**c**,**d**) The results of the same sources when they are separated by the distance of $D=\lambda$ which is not sub-wavelength. It is evident that under this circumstance, the generated image in the farfield can be resolved which is attributed to the fact that the diffraction limit has been obviated. (**e**,**f**) The near and far field results of the designed ASML with ANM when the sources are located at the sub-wavelength distance (i.e., $d=0.2 \lambda$). The obtained results are well abiding the attained results in (**c**,**d**).

### Numerical simulation

To demonstrate the capability of the proposed ASML based on the obtained materials of Eq. (4), several two dimensional (2D) numerical simulations were carried out by using COMSOL MULTIPHYSICS finite element solver. For all the propounded examples, we have assumed $h_1=0.2$, $h_2=0.99$ and $h_3=1$. The solving domain was considered as a $20\lambda \times 20\lambda$ square (where $\lambda$ is the wavelength corresponds to the operating frequency of 3 kHz), with perfectly matched layers (PML) locating at the ends of each side to absorb the outgoing waves from the interior of the computational region without reflecting them back into the solving domain. Moreover, hereafter, we will utilize a pair of line sources denoted by $S_1$ and $S_2$ that are separated by the sub-wavelength distance of $d=0.2 \lambda$ for all the given simulations. Since these two sources are located in a close vicinity of each other, their interfering pressure behaves similar to that of a single source (e.g., only $S_1$) due to the diffraction limit in both near and far field regions as shown in Fig. 2a,b, respectively. However, if the same sources are separated by a non-sub-wavelength distance of $D=\lambda$, then the effect of diffraction will be negligible and thus their images in the far-field region could be resolved properly as it is shown in Fig. 2c,d.

**Figure 3:**
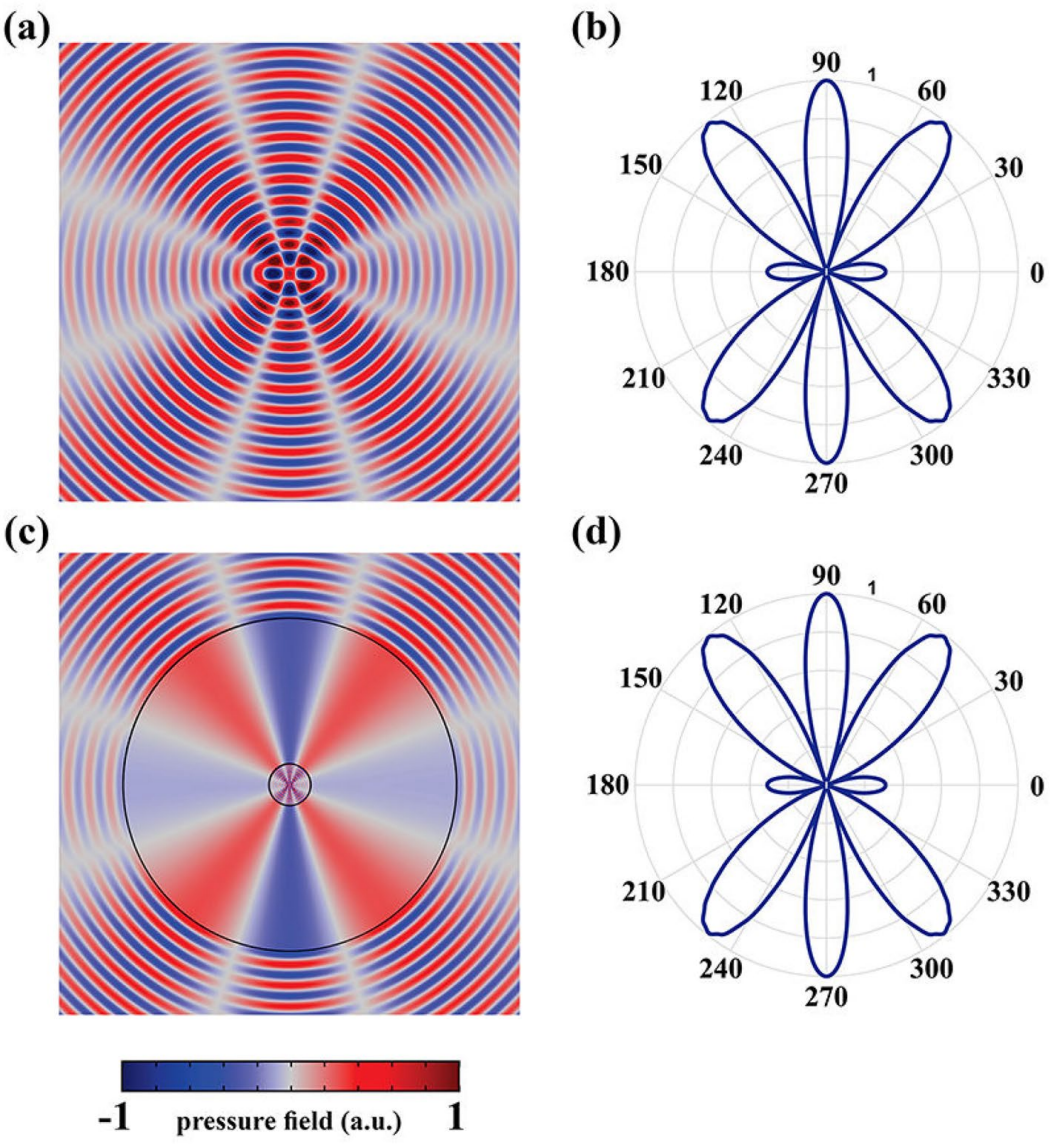
(**a**,**b**) The near and far field results of two line sources locating at the distance of $D=1.6\lambda$. (**c**,**d**) The pressure distributions of the same sources locating at the sub-wavelength distance after utilizing the ASML with new magnification factor. It should be remarked that the materials of the stretched region remain unchanged.

As it was mentioned previously, the goal is to design an ASML which is capable of producing exactly the same acoustic radiation pattern of the second case (i.e., when sources are separated by non-sub-wavelength distance of *D*), even though the sources are physically locating at sub-wavelength distance of *d*. This can also be viewed in the perspective of acoustic illusion, that is, we want to make an illusion for the outside viewer, in such a way that two acoustic sources separated by the distance of *d* in the physical space (shown in Fig. 2a), look like two sources locating at the distance *D* in the background medium. To this aim, firstly, we will design a cylindrical magnifying lens with the outer shape of $H(\varphi )=5\lambda$. By using the obtained materials of Eq. (4), the pressure distribution in near and far field regions will be obtained as they are shown in Fig. 2e,f. It is noteworthy to mention that selecting the exact values of $\infty$ and 0 for the mass-density components and bulk modulus causes numerical errors in the simulation process. To overcome this issue, we set $\Delta =0.001$. It is evident that the attained results are exactly the same as the case where sources are not separated by sub-wavelength distance. This indicates that the designed cylindrical shape magnifying lens is capable of resolving the image of the sources regardless of the diffraction limit.

**Figure 4:**
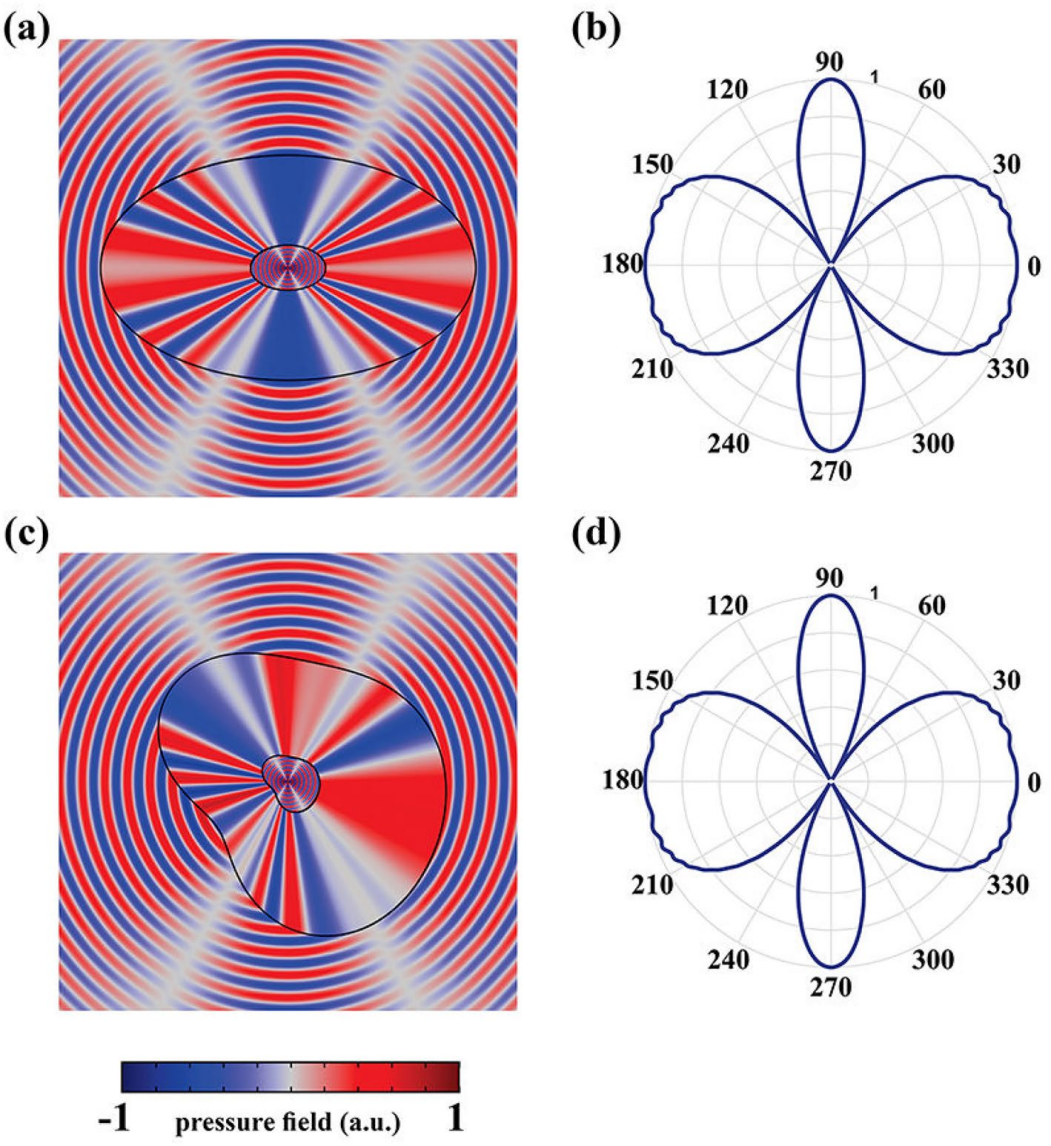
(**a**,**b**) The normalized near and far field results of the elliptical shape magnifying lens. (**c**,**d**) The same results of the same sources for a lens with an arbitrary shape cross-section. For both simulations, the material of the stretched region is the same as of the previous cases, which were obtained by considering $\Delta$ to be 0.001.

As it was discussed earlier, the ANM is independent of the device geometry. That is, if the radius of the coating layer is changed, there is no need to recalculate the demanding materials for the stretched region, which will subsequently give rise to reconfigurable magnification factor. To demonstrate this capability, we will set the desired distance of the sources to be $D=1.6\lambda$, as their pressure field distributions are shown in Fig. 3a,b, while their actual distance remains as $d=0.2\lambda$. This will lead to a magnification factor with the value of $\Gamma =D/d=8$. As it is depicted in Fig. 3c,d, by utilizing the same ANM that was used for the previous case (which its magnification factor was $\Gamma =5$), perfect functionality will be obtained in both near and far field regions, respectively. Furthermore, the competency of the proposed ANM is not restricted to any specific shape and; thus, it can be utilized for any desired geometry without degrading the performance of the ASML. This is of utmost importance for realistic problems since it might be necessary in some cases that the lens has an arbitrary cross-section. To the authors’ best knowledge, no systematic work has been yet proposed to overcome on this challenge. To demonstrate the capability of our method in designing an arbitrary shape magnifying lens, while the outer contour shape of the lens (i.e., $H(\varphi )$) alters in a manner that an arbitrary shape cross section is generated, we will use the same materials of Eq. (4) and the results of the pressure field in near and far fields region will be achieved as shown in Fig. 4.

As it is illustrated in Fig. 4, the near field pressure distributions are abide well with the case where sources are located at the non-sub-wavelength distance of $D=\lambda$ (shown in Fig. 2c). Moreover, their obtained far-field images are also identical to the case given in Fig. 2c, indicating that details of the object can be judiciously resolved from the designed ASML whose cross-sections are not restricted to the cylindrical shape.

## ASML implementation

As it was mentioned, to date, the inhomogeneous materials of the arbitrary shape magnifying lenses restrict their applicability for being used in realistic situations. In this section, we will show that the proposed ASML can be achieved using realistic structures. To this aim, firstly, the necessitating materials of Eq. (4) must be realized based on acoustics metamaterials. Afterwards, with the aid of effective medium theory (EMT) and the extracted parameters of the designed unit-cells, we will present a multilayered ASML that is capable of breaking the acoustics diffraction limit and resolves the image of the sources in the far field region. It should be mentioned that since we are solving the problem for 2D geometry, we can safely disregard the effect of $\rho _{33}$ in the mass density tensor. Therefore, the materials that must be realized for the ASML are $(\rho _c/\rho _0,\kappa _c/\kappa _0)=(1,0.04)$ for the compressed region and $\rho _s/\rho _0$ = diag$[0,\infty ]$and $\kappa _s/\kappa _0=\infty$, (diag$[\cdot ]$ represents a diagonal matrix) for the stretched region.

**Figure 5:**
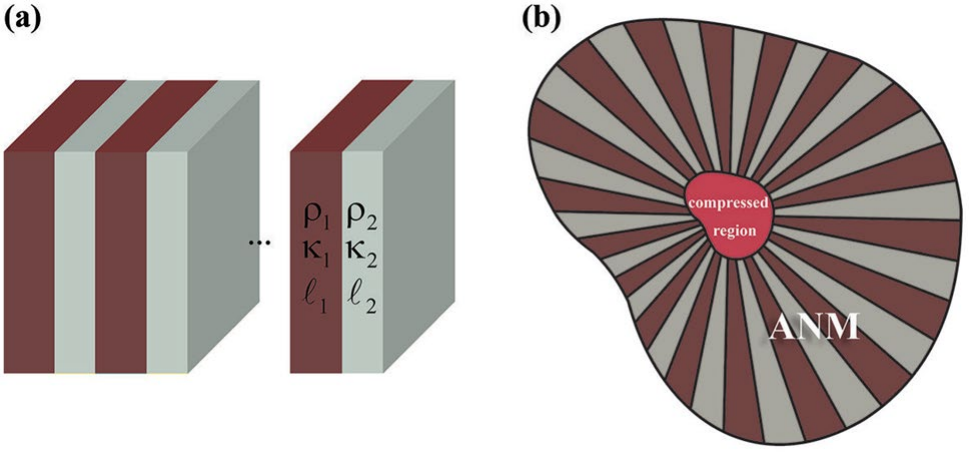
(**a**) A multi-layered system which mimics an anisotropic slab behavior. (**b**) The schematic of the final configuration of the realized structure.

According to EMT, it is known that the multi-layered structure shown in Fig. 5a, which its layers have the mass densities and bulk modulus of $\rho _1, \rho _2$ and $\kappa _1, \kappa _2$, respectively, can behave as an anisotropic medium with the parameters of Eq. (5), if the thickness of each layer (i.e., $l_1$ and $l_2$) is much smaller than the operating wavelength of $\lambda$.

5 $$\begin{aligned}&\rho _\phi = U\times \rho _1 + (1-U) \times \rho _2 ,\frac{1}{\rho _r}=\frac{U}{\rho _1}+\frac{1-U}{\rho _2} , \frac{1}{\kappa }= \frac{U}{\kappa _1}+\frac{1-U}{\kappa _2} \end{aligned}$$

$U=l_1/(l_1+l_2)$ is the filling fraction and the subscripts of $\phi$ and *r* indicate the direction transverse and along the propagation of acoustic waves, respectively as demonstrated schematically in Fig. 5b. According to the above-mentioned relation, the ANM has a mass density that is nearly zero along the $\hat{r}$ direction while at the same time it has a large value along $\hat{\varphi }$ direction.

**Figure 6:**
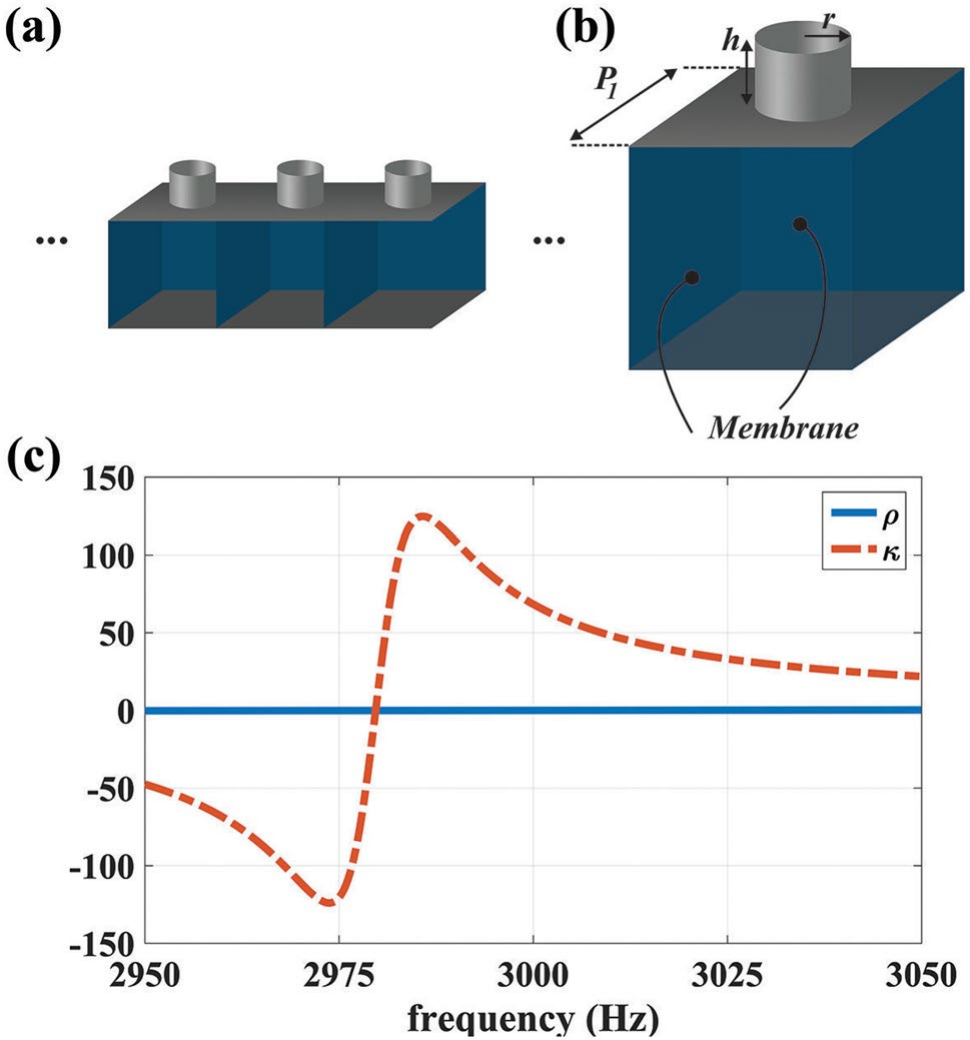
(**a**) The schematic illustration of side branch and membrane-based metamaterials. (**b**) The geometry of the designed meta-atom with the parameters of $h=2.36$ mm and $r=0.4$ mm. (**c**) The retrieved relative mass density and bulk modulus extracted from full-wave simulations.

**Figure 7:**
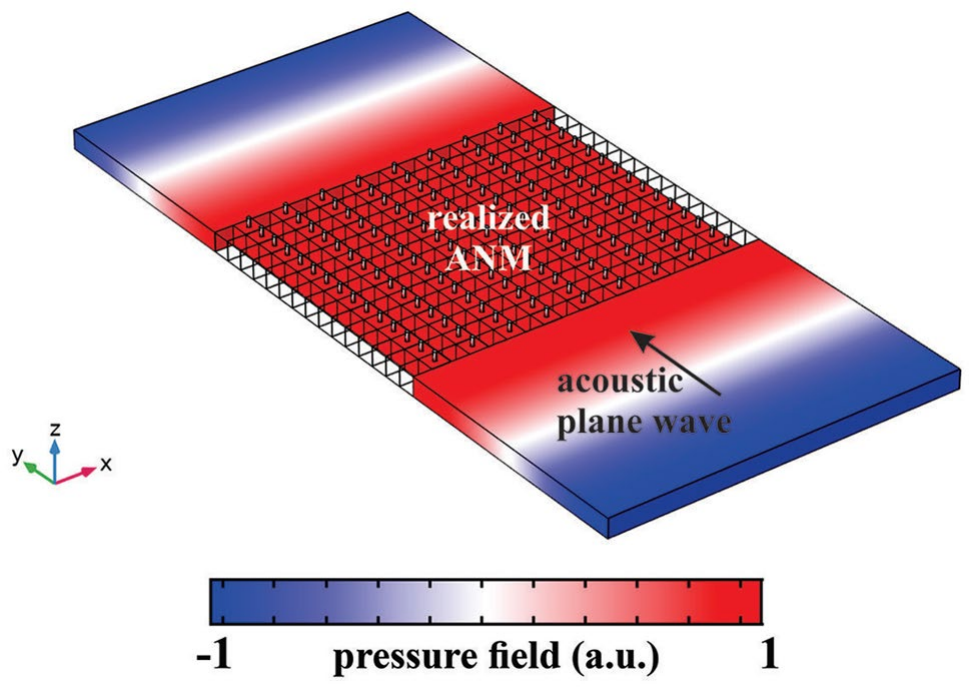
The behavior of the realized ANM under the incidence of an acoustic plane wave. The incident plane wave on the input boundary is perfectly transmitted to the output interface without distortion.

To realize such a property based on Eq. (5), the constructing layers of Fig. 5a could be considered to have a same thickness (i.e., $l_1=l_2$) while their acoustic parameters are set to ${{\rho }_{1}}=\infty ,\,{{\kappa }_{1}}=\infty$ and ${{\rho }_{2}}=0,\,{{\kappa }_{2}}=\infty$ with respect to the background medium. Therefore, the first layer (filled with $\rho _1=\infty$ and $\kappa _1=\infty$) can be easily replaced by a rigid wall boundary condition (RWBC) or filled with metals such as brass, stainless steel, and silver. It should be emphasized that the choice of RWBC or an actual metallic material mainly affects the simulation time and has negligible effect on the final results. As the second layer has arbitrary acoustic parameters (i.e., $\rho _2=0$ and $\kappa _2=\infty$), a quasi two-dimensional non-resonant meta-atom should be utilized to realize its parameters as it is shown in Fig. 6a,b^37^. As it is schematically illustrated in these figures, the meta-atoms consist of elastic membranes and side branches with open ends. The proposed configuration offers the advantage of controlling mass density and bulk modulus independently, where the mass density is solely controlled by the membranes and the bulk modulus is merely dependent on the side branches^38^. The membrane material is selected to be aluminum with Young modulus of 70 GPa, the Poisson ratio of 0.33 and the mass density of 2700 kg/m$^3$; and the tension on the membrane is assumed to be zero. The membrane size and thickness are set to be 4 mm $\times$ 4 mm and $5.892\,\upmu$m, respectively which lead to near zero mass density. To have a high value bulk modulus, the dimensions of the side branches are considered as $h=2.36$ mm and $r=0.4$ mm. The determinant acoustic parameters will be achieved with the retrieval algorithm^37^ as they are depicted in Fig. 6c, where the relative mass density and bulk modulus are obtained as 0.02 and 68, respectively. It should be mentioned that although the retrieved parameters of the proposed unit cell is not exactly the same as that of Eq. (7b), they can approximately mimic the behavior of an ideal ANM. To verify the functionality of the realized ANM, a three-dimensional (3D) numerical simulation is performed via COMSOL MULTIPHYSICS where the acoustic plane wave impinges on a slab consisting of multilayered structures filled with metal and the proposed meta-atoms and its results are shown in Fig. 7.

**Figure 8:**
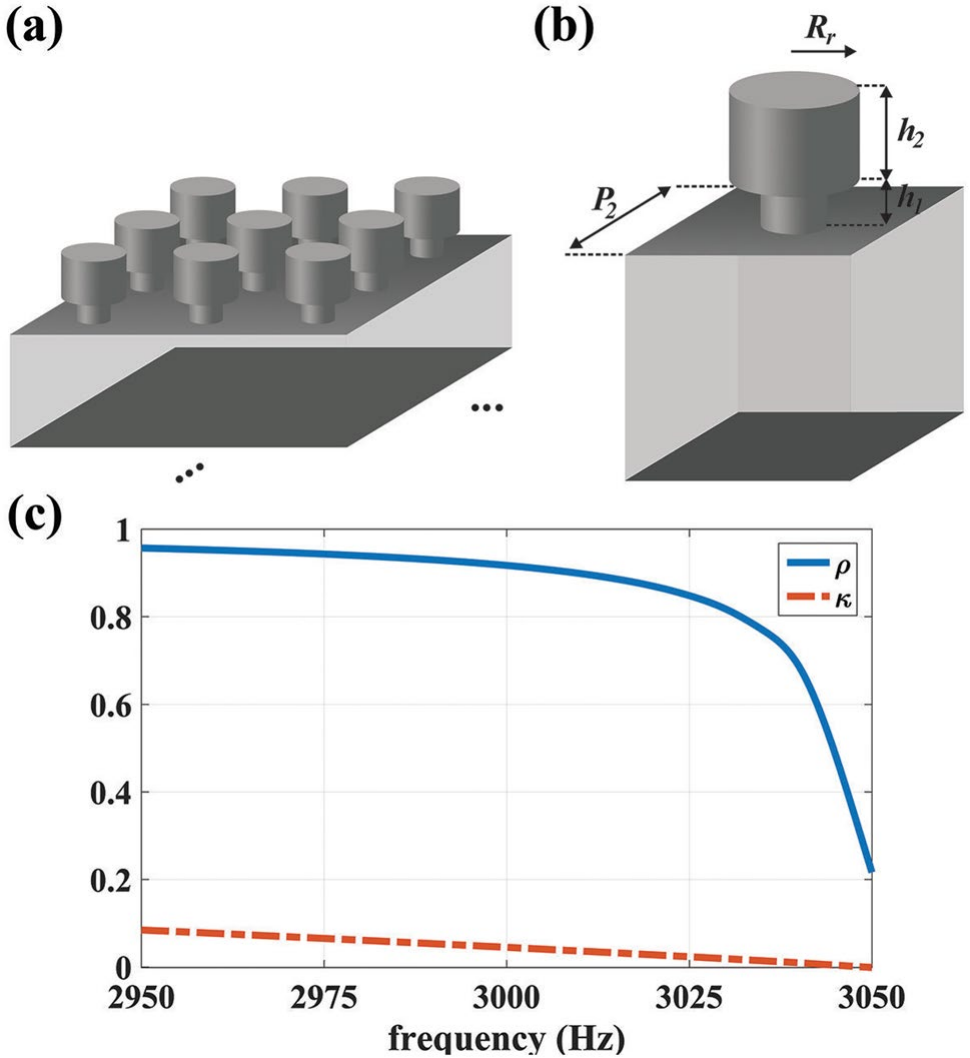
(**a**) The two dimensional schematic of Helmholtz resonator metamaterials. (**b**) The structural demonstrations of the realized unit cell with the parameters of $P_2=4$ mm, $h_1=1$ mm, $h_2=6.38$ mm and $R_r=1.5$ mm. (**c**) The retrieved relative mass density and bulk modulus extracted from full-wave simulations.

As can be seen in Fig. 7, the acoustic fields will be mapped point to point from the front face to the back one, which validates the correctness of the designed ANM^26^.It should be mentioned that since the utilized materials do not have any inherent loss and as the structure itself does not posses any resonance behavior, the loss effect is significantly small and it is safe to disregard it.

**Figure 9:**
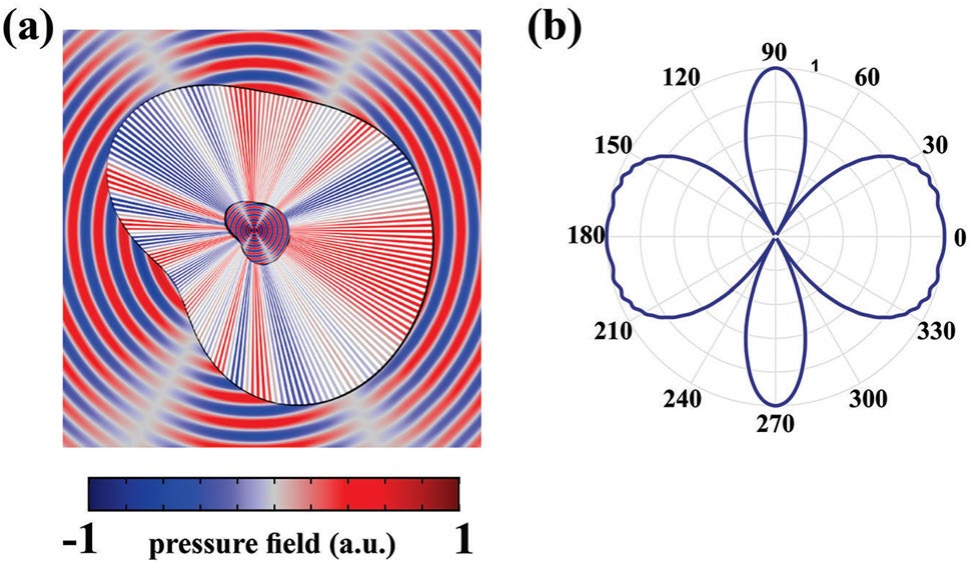
(**a**) The pressure field of an ASML realized with the materials extracted from proposed acoustics metamaterials. (**b**) Its corresponding far field distributions. The farfield result clearly demonstrate the retrieval of the image sources.

Besides the stretched region, to implement the materials of the compressed domain with near zero bulk modulus, an arrays of repeated unit cells with shunted Helmholtz resonators (HR) are exploited which are demonstrated in Fig. 8a^39^. The unit cells are made of metal (alternatively they could be assumed as rigid wall boundary condition in the simulations) By adjusting the geometrical features of HR which are schematically shown in Fig. 8b, the required acoustic parameters can be obtained as they are shown in Fig. 8c where the desired bulk modulus is attained at 3 kHz. Therefore, according to the extracted materials given in Figs. 6c and 8c, and by taking advantages of EMT which is schematically shown in Fig., an ASML has been implemented and its results are depicted in Fig. 9. It should be remarked that the number of the utilized layers to implement an ASML depends on the computational power of the system. In particular, the more the layers are increased, the more accurate the final results will be. However, this will impose constraints on the meshing of the whole structure. According to such a trade off, we have selected the number of the consisting layers for the stretched layer to be 360. In other words, we utilized 360 layers with equal thickness and then applied the retrieved parameters of Fig. 6 and RWBC to their corresponding slabs, while for the compressed region, we simply used the retrieved material of Fig. 8. As can be seen from Fig. 9a, the near field pressure distribution of the two sources located at the sub-wavelength distance (i.e., $d=0.2 \lambda$) surrounded by the implemented lens is almost the same as that of the case of Fig. 4c which an ideal ANM has been used. Moreover, the corresponding far field results of the realized lens is also similar to the case of Fig. 4d indicating that the proposed lens can effectively break the diffraction limit and the resolves the images of the exploited sources in the far field region.

## Discussion

In conclusion, we have proposed a method to design an ASML, which is capable of obviating the diffraction limit. The obtained materials through the propounded approach became ANM which are homogeneous and independent of the device geometry. This will consequently enable the proposed ASML to be capable of magnifying arbitrary shape regions without the demand of recalculating or redesigning the necessitating material. Moreover, owing to this feature of ANM, the proposed ASML can have reconfigurable magnification factor, which is of utmost importance in practical scenarios. The ANM is implemented with two acoustic meta-atoms named HR and cubic blocks with clamped elastic membranes and side branches. Then, as a proof of concept, an ASML was realized based on the retrieved parameters from the designed unit cells and with the aid of EMT. It was observed that the realization results were well abide with the theoretical predictions and numerical simulations. The propounded ASML can pave the way towards many functionalities and applications such as medical imaging, as well as focused ultrasound surgery.

## Methods

### Derivation of ANM

As it was mentioned, setting ${{h}_{2}}\rightarrow {{h}_{3}}$ will yield the acoustic wave to be independent of $\rho _{12}$ and $\rho _{21}$. To demonstrate this mathematically, the acoustic wave equations (i.e., $\bar{\bar{\rho {^\prime} }}\times \partial \mathbf{v} /\partial t=- \nabla P$ and $\partial P/ \partial t =-\kappa {^\prime} \times \nabla \cdot \mathbf{v}$) in a medium defined by $\rho _s{^\prime}$ and $\kappa _s{^\prime}$ should be derived by substituting Eq. (2) in the corresponding equations as

6 $$\begin{aligned}&\rho _{22} \frac{\partial ^2 P}{\partial r^2 }+\rho _{22} \frac{\partial P}{\partial r }- 2\rho _{12} \frac{\partial ^2 P}{\partial r \partial \varphi } + \frac{\rho _{11}}{r}\frac{\partial ^2 P}{\partial \varphi ^2 } -\omega ^2 (\frac{\rho ^2_{12}-\rho _{11}\rho _{22}}{\kappa {^\prime} })P=0 \end{aligned}$$

On the other hand, by assuming conformal boundaries together with setting $h_2 \rightarrow h_3$, the materials of the stretched region will be changed to more simplified ones as

7 $$\begin{aligned} \bar{\bar{\rho _s{^\prime} }}= \begin{bmatrix} \Delta &{} \frac{dH(\varphi )/d\varphi }{H(\varphi )} &{} 0 \\ \frac{dH(\varphi )/d\varphi }{H(\varphi )} &{} 1/\Delta &{} 0 \\ 0&{} 0&{} 1/\Delta \end{bmatrix} , \kappa _s{^\prime} = 1/\Delta \end{aligned}$$

where $\Delta = 0$. Therefore, by substituting Eq. (7) into Eq. (6), the governing acoustic wave propagation will be obtained as

8 $$\begin{aligned}&\frac{\partial ^2 P}{\partial r^2} + \frac{\partial P}{\partial r}-2\{ \Delta \times \rho _{12}\} \frac{\partial ^2 P}{\partial r \partial \varphi } + \frac{\Delta ^2}{r}\frac{\partial ^2 P}{\partial \varphi ^2} -\omega ^2 (\rho ^2_{12}- 1)\times \Delta ^2 \times P =0 \end{aligned}$$

However, as $\Delta = 0$ and $\rho _{12}$ has a finite value according to Eq. (7), the exact value of $\rho _{12}$ is not important because only the product of these values plays crucial role in the obtained wave propagation equation not each of them individually. Hence, without the loss of generality, one can assume any desirable finite value for these off-diagonal components. For this purpose, we have assumed that $\rho _{12}=0$.

### Dispersion relation

To further gain insight about the ANM, the dispersion relation in such a medium will be investigated. It is known that the dispersion expression for an anisotropic mass density can be expressed as^40^

9 $$\begin{aligned} \frac{k_r^2}{\rho _{11}}+\frac{k_{\varphi }^2}{\rho _{22}}=\frac{\omega ^2}{\kappa } \end{aligned}$$

where $\omega$ is the frequency and $k_r$ and $k_\varphi$ are the wave-vector components along the radial and azimuthal directions, respectively. Substituting Eq. (4b) in the dispersion relation, it is evident that as $\rho _{22}= \infty$, $k_\varphi$ could take any value, implying that $k_\varphi$ does not have any influence on the wave propagation inside an ANM medium, and thus can be safely neglected. Meanwhile, the radially wave number in the ANM medium will be zero as $k_r=\omega \sqrt{\rho _{11}/\kappa } = 0$ and subsequently will result in no propagation in the radial direction. Therefore, as $k_\varphi$ does not effect the wave propagation and $k_r$ has zero value, the acoustic fields would be mapped point-to-point from the inner boundary to the outer one and vice versa under the utilization of ANM. It is notable to mention that since selecting the exact values of $\infty$ and 0 for the ANM material is impossible, instead of having $\Delta = 0$ we have assumed that $\Delta \rightarrow 0$. This is a rational assumption since it still yield the wave-vector along the radial direction to be zero (i.e., $k_r=\omega \sqrt{\rho _{11}/\kappa }= \omega \times \Delta \rightarrow 0$) and, therefore, we can yet suppose that the acoustic fields are mapping from the outer boundary to the inner one point-to-point.
